# Editorial: Applications of Nanobiotechnology in Pharmacology

**DOI:** 10.3389/fphar.2019.01451

**Published:** 2019-12-04

**Authors:** Baowen Qi, Chao Wang, Jianxun Ding, Wei Tao

**Affiliations:** ^1^Center for Nanomedicine and Department of Anesthesiology, Brigham and Women’s Hospital, Harvard Medical School, Boston, MA, United States; ^2^Jiangsu Key Laboratory for Carbon-Based Functional Materials and Devices, Institute of Functional Nano & Soft Materials (FUNSOM), Soochow University, Suzhou, China; ^3^Key Laboratory of Polymer Ecomaterials, Changchun Institute of Applied Chemistry, Chinese Academy of Sciences, Changchun, China

**Keywords:** pharmaceutical nanotechnology, drug delivery, nanomedicine, theranostics, biomaterial, regenerative medicine

Nanobiotechnology, by definition, is a multi-strategic technique that combines nanotechnology and biotechnology to engineer the properties of therapeutic agents, e.g., target delivery of therapeutics by nanoparticles, in a unique way as paradigm shifts from fundamental biological study to clinical pharmacology. A well-defined nanosystem has controllable dimensions and properties being able to carry various functional biomolecules, such as small molecules, proteins, genes, and so forth. Those unusual characteristics enable them to exhibit prominent efficacies for the diagnostic and/or treatment of numerous diseases like cancer *via* precisely tuning the size, morphology, and surface property. Moreover, strategies to achieve a better therapeutic purpose rely on “responsive” nanomaterials that release the active substances under specific stimuli, such as pH, redox potential, temperature, enzymes, or other external stimuli dependent on their unique physicochemical conditions. It is particularly noteworthy that the synergistic combination of nanoparticles with different target ligands facilitates the development of more efficient “active” drug delivery systems.

Furthermore, the sensitivity of *in vivo* real-time diagnosis can be enhanced by combing nanotechnology with a contrast agent for next-generation precision medicine. However, the safety issues of nanosystems as well as their clinical efficacies remain controversial, which concern intrinsically originated from four aspects: 1) synthetic nanoparticles are generally composed of inorganic or organic materials, which may trigger cytotoxic pathways; 2) nanoparticles alters the biodistribution of the carried agents, which may, in turn, change the toxicological behaviors of the agents as well; 3) the enhanced permeability and retention (EPR) effect-based tumor-targeting nanomedicines is hard to be proved in humans, which was explained by the drastically difference between murine and human tumor tissues; 4) the complicated protein coronas on the surface of nanoparticles disturbs their metabolism behaviors. Although there is still a fierce debate over the fate of this emerging field, we still hold very optimistic attitudes toward the revolution of nanobiotechnology brought to the future pharmacological realms.

In the current topic, an overview of applications of nanobiotechnology in pharmacology is provided through 26 articles by 206 authors, which contains 2 reviews and 24 original research papers (Total views: 40,096; as of Oct 1^st^, 2019). One of the reviews summarized the currently available knowledge on the role of clinically approved poly(ethylene glycol)–polylactide (PEG–PLA) copolymer micelles as nanocarriers for therapy of malignancies (Wang et al.). Another review summarized the studies using carbon-based nanomaterials, including carbon nanotubes, graphene oxide (GO), and graphene quantum dots, being extensively investigated for various applications, e.g., biosensing and cancer therapy, owing to their structural diversities (Maiti et al.).

The special topic contains many original studies covering critical aspects of nanobiotechnology. Among them, a few self-assembled nanoparticles were meticulously designed and prepared using biocompatible polymers. To start with, PEG as a hydrophilic polymer is widely used for self-assembly with other sets of hydrophobic components to form “core–shell” nano-structures, for instance, PEG-based platinum(II) (Pt(II)) nanoformulation was developed to combat multidrug-resistant cancer (Tsai et al.). Similarly, PEG–PLA nanocarrier containing both Pt(IV) and capecitabine was developed using a co-assembly approach (Xiao et al.). Self-assembled nanoparticle prepared by the conjugation PEG to β-cyclodextrin (PEG-CD) was able to efficiently deliver doxorubicin (DOX) and sorafenib in a rational manner (Xiong et al.), and a multi-functional peptide-modified PEG-CD complex was developed for diabetes and immunotherapy (Dong et al.). PEG-poly(ε-caprolactone) (PEG-*b*-PCL) was able to deliver cytokines in order to prohibit the migration of breast cancer cells (Liang et al.) and PEG–poly(amino acid) was effectively used to deliver DOX as a benefit of the EPR effect (Niu et al.).

Pluronic F127 is a triblock copolymer composed of poly(ethylene glycol)–poly(propylene glycol)–poly(ethylene glycol) (PEG–PPG–PEG). The hydrophobic drug retinoic acid was stabilized with Pluronic F127 to form micelle for combination therapy against solid tumor (Zhu et al.). In order to enhance the efficacy of antitumor drug docetaxel (DTX), aptamer-polydopamine-functionalized nanoparticle was prepared and utilized to enhance chemo-photothermal therapy for breast cancer (Kong et al.), and hydrophobic poly(ester amide) nanoparticle was used to encapsulate DTX for the suppression of non-small-cell lung cancers (Chen et al.).

Chitosan (CS), hyaluronic acid (HA), and polygalactose are polysaccharide biomaterials, which are also discussed in this special issue. For instance, CS oligosaccharide was used to inhibit the intrinsic coagulation pathway due to its biocompatible activity (Guo et al.). A natural product quercetin was encapsulated into CS nanoparticle as a potential therapeutic agent for topical application against ultraviolet B radiation (Nan et al.). Hydrophilic HA can specifically target CD44 on the cancer cells, which was able to self-assemble with hydrophobic photosensitizer chlorin e6 (Ce6) to form into a micelle. The micellar system demonstrated a redox-responsive kinetics to controllably release of payloads for the enhancement efficacy of photodynamic therapy (PDT) (Feng et al.). Besides, glycopolymer formed nanoparticle by crosslinking with the hydrophobic drug, therefore possessing both redox-responsive and pH-sensitive characteristics for precise hepatoma therapy (Wu et al.).

In addition to polymer nanoparticles, liposomes or lipid nanoparticles also played vital roles in pharmacology. One study was to encapsulate brinzolamide into hydropropyl-β-cyclodextrin inclusion complex and was then formulated as nanoliposome by the thin-film dispersion method (Wang et al.). In the same research group, the authors synthesized mannose-cholesterol conjugate by click reaction with PEG of different molecular weights to prepare another unique nanoliposome (Wang et al.). Moreover, encapsulating peptide into lipid nanoparticle possessed both the superior nature of polymer nanoparticle and liposome. Therefore a high oral bioavailability and sustained release kinetics were achieved (Zhao et al.).

Nowadays, inorganic materials also demonstrated high potentials to be used in pharmacology. The superparamagnetic iron-oxide nanoparticle was attractive due to its unique properties to deliver paclitaxel, offering both magnetic targeting and receptor-mediated targeting outcome (Wang et al.). The ultra-small nano-ceramide-GO nanoparticle was devised for treating hepatoma (Wang et al.). Polymer-coated black phosphorus nanosheet showed long circulation kinetics and an excellent cell uptake capacity *in vivo*, providing a synergistic cancer therapeutic strategy (Gao et al.).

Overall, this research topic discussed a few proofs of concept by taking the advantages of nanobiotechnology in pharmacology. Such an emerging field provided insightful thought from fundamental nanotechnology researches to translational medicine, meanwhile presenting a future perspective to achieve the success of nanotechnology as a return.

## Author Contributions

BQ, CW, JD and WT contributed to writing this Editorial.

## Conflict of Interest

The authors declare that the research was conducted in the absence of any commercial or financial relationships that could be construed as a potential conflict of interest.

